# An Amphiphilic Multiblock Polymer as a High-Temperature Gelling Agent for Oil-Based Drilling Fluids and Its Mechanism of Action

**DOI:** 10.3390/gels9120966

**Published:** 2023-12-09

**Authors:** Yinbo He, Mingliang Du, Jing He, Haiyang Liu, Yanhua Lv, Lei Guo, Peng Zhang, Yunhai Bai

**Affiliations:** 1 National Key Laboratory of Petroleum Resources and Engineering, China University of Petroleum (Beijing), Beijing 102249, Chinalhy1475084866@163.com (H.L.);; 2 MOE Key Laboratory of Petroleum Engineering, China University of Petroleum (Beijing), Beijing 102249, China; 3 China Oilfield Services Ltd., Langfang 065201, China; 4 Drilling & Production Technology Research Institute, CNPC Chuanqing Drilling Engineering Company Ltd., Xi’an 710129, China

**Keywords:** gelling agent, oil-based drilling fluid, weak gel, high-temperature resistance

## Abstract

Oil-based drilling fluids are widely used in challenging wells such as those with large displacements, deepwater and ultra-deepwater wells, deep wells, and ultra-deep wells due to their excellent temperature resistance, inhibition properties, and lubrication. However, there is a challenging issue of rheological deterioration of drilling fluids under high-temperature conditions. In this study, a dual-amphiphilic segmented high-temperature-resistant gelling agent (HTR-GA) was synthesized using poly fatty acids and polyether amines as raw materials. Experimental results showed that the initial decomposition temperature of HTR-GA was 374 °C, indicating good thermal stability. After adding HTR-GA, the emulsion coalescence voltage increased for emulsions with different oil-to-water ratios. HTR-GA could construct a weak gel structure in oil-based drilling fluids, significantly enhancing the shear-thinning and thixotropic properties of oil-based drilling fluids under high-temperature conditions. Using HTR-GA as the core, a set of oil-based drilling fluid systems with good rheological properties, a density of 2.2 g/cm^3^, and temperature resistance up to 220 °C were constructed. After aging for 24 h at 220 °C, the dynamic shear force exceeded 10 Pa, and G′ exceeded 7 Pa, while after aging for 96 h at 220 °C, the dynamic shear force exceeded 4 Pa, and G″ reached 7 Pa. The synthesized compound HTR-GA has been empirically validated to significantly augment the rheological properties of oil-based drilling fluids, particularly under high-temperature conditions, showcasing impressive thermal stability with a resistance threshold of up to 220 °C. This notable enhancement provides critical technical reinforcement for progressive exploration endeavors in deep and ultra-deep well formations, specifically employing oil-based drilling fluids.

## 1. Introduction

Drilling fluids colloquially deemed the “blood” of drilling operations, fulfill quintessential roles within drilling engineering [1]. They are instrumental in cooling and lubricating drilling apparatus, fortifying wellbore structures, equilibrating reservoir pressure, and effectuating the transport and suspension of drilling cuttings [2]. These fluids are broadly classified into water-based, oil-based, and gas-based categories, contingent upon their dispersion medium [3,4]. Oil-based drilling fluids, typified by a brine dispersal phase and a continuous phase of base oils such as mineral, diesel, or synthetic oils—amalgamated with apt emulsifiers, wetting agents, gelling agents, and density enhancers—constitute a robust oil-in-water emulsifying system [5,6,7,8,9]. Despite their less prevalent utilization compared to water-based variants, oil-based drilling fluids are distinguished by their enhanced wellbore stability, inhibition efficacy, lubricative properties, and thermal fortitude, rendering them the preferred option for drilling in unconventional reservoirs, intricate geological structures, and extreme temperature and pressure conditions [10,11,12].

Rheological parameters are pivotal in evaluating the performance of oil-based drilling fluids, mirroring their proficiency in mobilizing and sustaining solids like drill cuttings and barite. These parameters encompass plastic viscosity, yield point, dynamic plastic ratio, storage modulus (G′), loss modulus (G″), and low shear rate viscosities (Ф6, Ф3) [13,14,15]. Plastic viscosity is indicative of internal resistance to fluid motion, where elevated levels may escalate drilling pressure losses and impede efficient hydraulic conditions, thus deterring rapid drilling operations. The yield point signifies the fluid’s capacity to establish a spatial network conducive to carrying cuttings at reduced annular velocities. The dynamic plastic ratio quantifies the fluid’s shear-thinning behavior. The moduli G′ and G″ gauge the integrity of the fluid’s structural network under quiescent states, with higher values favoring the suspension of solids. Nonetheless, an overly robust network may complicate pumping resumption. The low shear rate viscosities, Ф6 and Ф3, are crucial for the steady carriage of solids within the fluid matrix [16,17]. Conventionally, enhancement of the rheological attributes of oil-based drilling fluids is pursued through a gelling agent, aimed at bolstering fluid viscosity and yield stress while concurrently mitigating the impact on plastic viscosity. These gelling agents are pivotal in amplifying drilling fluid viscosity and yield stress, thereby sustaining their functionality in suspending and transporting drill-related particulates.

In the ambit of contemporary advancements in ultra-deep hydrocarbon exploration, breakthroughs have been chronicled globally, with notable discoveries within China’s Tarim and Sichuan Basins, where reservoirs surpassing depths of 8000 m have been encountered [18,19]. These explorations have unveiled reservoirs with escalating temperature profiles, thereby catalyzing a spectrum of both challenges and prospects for oil-based drilling fluids. Contemporary formulations of such fluids exhibit pronounced limitations in rheological behavior when subjected to elevated thermal conditions. For instance, high-temperature exposure often precipitates a marked diminution in both viscosity and yield stress, compromising the fluid’s competency in suspending and conveying dense minerals, cuttings, and particulates. This phenomenon can precipitate complications such as sedimentation and incidents of pipe obstruction. Moreover, drilling fluids may undergo excessive thickening at elevated temperatures, precipitating wellbore instability, operational difficulties such as pump restarts, and other malfunctions [20,21,22,23]. Subsequently, the imperative to innovate and formulate a gelling agent, resilient to high temperatures, becomes evident. Such an agent would surmount the rheological tribulations posed to oil-based drilling fluids, thereby bolstering the safe and efficient exploitation of ultra-deep hydrocarbon reserves.

The scholarly and industrial sectors have delved into gelling agents suitable for oil-based drilling fluids, yielding a diversity of inventions including organic clay, nano-composite, and polymer-based gelling agents. Organic clay, a prevalent thickening agent derived from the modification of bentonite with quaternary ammonium cations, serves to augment the microstructure at the oil-water interphase via adsorption, thereby ameliorating the rheological properties of the drilling fluids. Yet, such clays exhibit a propensity for desorption and thermal degradation of their modifying agents beyond 180 °C, resulting in escalated plastic viscosity and undue fluid thickening post-high-temperature exposure. This deficiency constrains their utility in high-temperature drilling scenarios [24,25,26]. Distinguished researchers such as Noah A. Z. have contrived a polymer/nano-composite gelling agent amalgamating zinc oxide nanoparticles, modified calcium carbonate nanoparticles, and polystyrene-butadiene rubber (PSBR) copolymers [27]. This formulation has demonstrated an enhancement in the yield point of oil-based drilling fluids, albeit with a performance ceiling of 150 °C [28,29]. Oil-soluble polymer gelling agents, manifesting high molecular weight, construct a network within drilling fluids primarily through molecular entanglement or adhesion to solid-phase entities [30,31]. Despite their effectiveness, these agents are susceptible to molecular degradation at elevated temperatures, which could culminate in structural failure and an inadvertent surge in plastic viscosity, deterring rapid drilling progression. In contrast, fatty acid amide gelling agents, synthesized via the amidation of fatty acids with organic amines, harbor thermal stability attributable to their amide linkages, endowing them with resistance to hydrolytic degradation under thermal duress. Currently capable of enduring temperatures nearing 180 °C, these agents harbor significant potential for advancement and application in high-temperature drilling operations.

In the nascent phase of this investigation, a small-molecule gelling agent was synthesized leveraging dibasic acid and diethanolamine as precursors, illustrating that the employment of amphiphilic small-molecule polymers for modulating emulsion interfacial film characteristics constitutes an efficacious strategy for fabricating a spatial network matrix [32]. Nevertheless, such gelling agents exhibit a confined number of adsorptive moieties, rendering them suboptimal for the rheological modulation of oil-in-water emulsions, particularly when juxtaposed with high-molecular-weight counterparts. High-molecular-weight polymers, while beneficially impacting the rheological attributes of oil-based drilling fluids, inadvertently escalate plastic viscosity and exhibit diminished resistance to high thermal conditions. Ergo, the current study propounds the design of an amphiphilic segmented low-molecular-weight polyether fatty acid amide gelling agent, specifically for oil-based drilling fluid with high-temperature resistance. The molecular architecture of low-molecular-weight polymers mitigates susceptibility to thermal-induced chain scission and degradation, phenomena predominantly associated with high-molecular-weight polymers. Their diminutive molecular stature and attenuated internal frictional forces engender only a nominal rise in plastic viscosity. The amphiphilic nature of these polymers permits adsorption at the oil-water interphase within emulsions, thereby facilitating the modulation of droplet interfacial films and emulsion aggregation morphologies. The segmented configuration amplifies the array of adsorptive sites, thereby augmenting the agent’s capacity to influence emulsion rheology.

This manuscript delves into the rheological control mechanism wielded by fatty acid amide gelling agents within oil-based drilling fluids and addresses the enhancement of polymer thermal stability. Polyfatty acids, selected for their intrinsic oleophilicity and partial hydrophilicity, serve as the monomeric foundation, while the incorporation of ether linkages bolsters hydrophilicity, culminating in the formation of a segmented polymer, designated as HTR-GA. This polymer is embodied by amide functionalities, ether segments, and elongated aliphatic chains. HTR-GA’s physicochemical properties were elucidated through Fourier-transform infrared spectroscopy (FTIR) and thermogravimetric analysis (TGA). The discourse evaluates the rheological ramifications of HTR-GA on pristine emulsions, emulsions compounded with organic clay, and comprehensive oil-based drilling fluid systems, concurrently unveiling its modulatory mechanism on emulsion rheology.

## 2. Results and Discussion

### 2.1. Characterization of HTR-GA

#### 2.1.1. FT-IR

**Figure 1:**
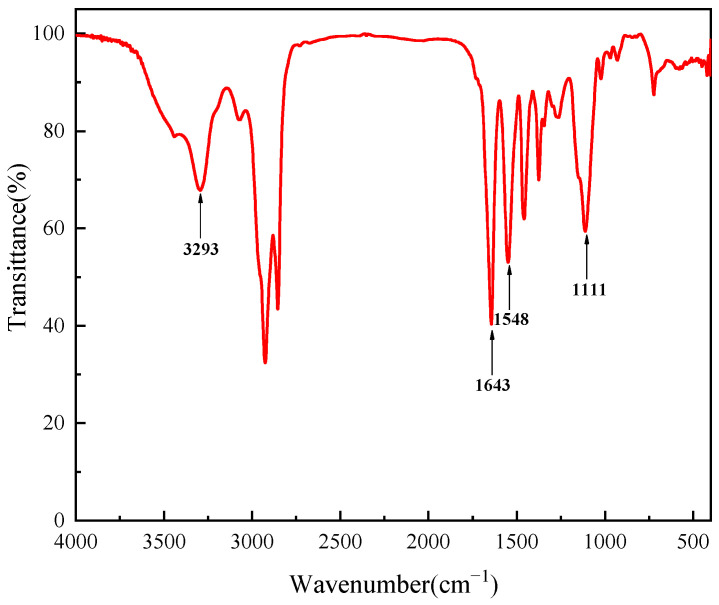
Infrared spectra of HTR-GA.

The infrared spectroscopy analysis provided crucial molecular insights into HTR-GA, as depicted in Figure 1. The absence of a distinct carboxylic acid C=O stretching peak within the 1700–1750 cm^−1^ range and the presence of characteristic bands N-H stretching at 3293 cm^−1^, C-O-C antisymmetric stretching at 1111.84 cm^−1^, and C=O stretching of the amide linkage at 1643.42 cm^−1^, alongside N-H bending at 1548.99 cm^−1^ corroborate the formation of the amide functional group. These spectral features are indicative of the amidation reaction’s success, asserting the efficacy of the synthetic process employed to produce HTR-GA. The FT-IR results thus validate the chemical structure of the synthesized HTR-GA, confirming its potential utility as a high-temperature-resistant additive for oil-based drilling fluids.

#### 2.1.2. TGA

**Figure 2:**
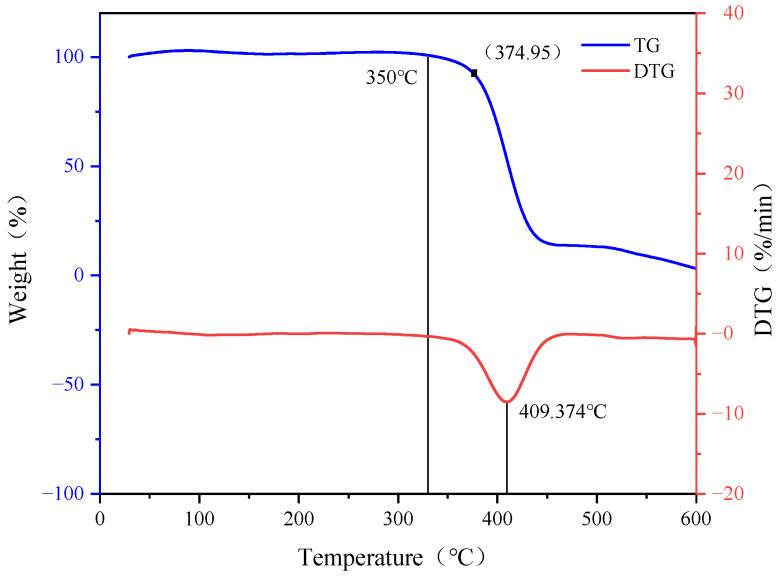
Thermogravimetric curve of HTR-GA.

The thermogravimetric profile of HTR-GA, detailed in Figure 2, demonstrates the polymer’s commendable thermal endurance. Notably, HTR-GA exhibits negligible weight loss until reaching the critical temperature of 374 °C, underscoring its resistance to high-temperature-induced degradation, a characteristic often compromised in high molecular weight counterparts. The observed stability is attributed to robust intermolecular forces, particularly hydrogen bonding among amide and ether groups, which fortify the molecular architecture against thermal stress. The onset of thermal decomposition is registered beyond 374 °C, with the most pronounced loss at 409 °C, leading to a substantial reduction in mass by 420 °C. The residue post complete degradation at 599.8 °C stands at 3.11%. The experiment, performed under a nitrogen atmosphere, indicates that HTR-GA’s thermal cracking threshold aptly demonstrates its potential for high-temperature applications.

#### 2.1.3. GPC

The molecular weight characterization of the synthesized HTR-GA is shown in Table 1. Analysis of the data presented in Table 1 reveals that the HTR-GA exhibits a number-average molecular weight (M_n_) of 19,547 Daltons (Da), a weight-average molecular weight (M_w_) of 40,263 Da, a peak molecular weight (M_p_) of 37,201 Da, and a z-average molecular weight (M_z_) of 66,916 Da. These molecular weight metrics collectively indicate that the synthesized HTR-GA is characterized as a polymer of low molecular weight.

### 2.2. Impact of HTR-GA on Emulsion Stability

The surfactant-like molecular structure of HTR-GA, characterized by hydrophilic polyether and hydrophobic fatty acid chains, inherently seeks the oil-water interface, influencing emulsion stability—a function of interfacial tension and membrane strength. Stability assessments through emulsion-breaking voltage measurements reveal that HTR-GA enhances stability, as evidenced by increasing voltage values correlating with increased HTR-GA concentrations.

**Figure 3:**
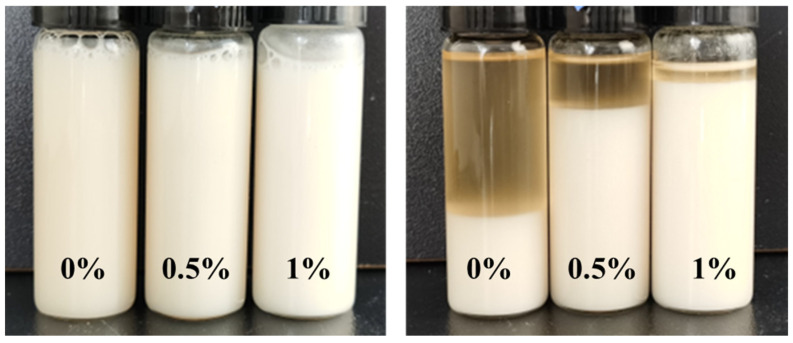
Influence of HTR-GA on the stability of Oil-in-Water emulsions.

**Figure 4:**
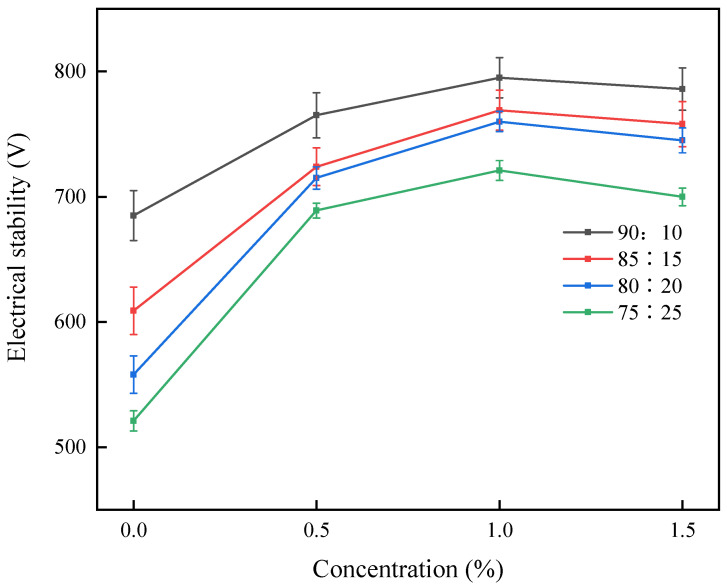
Effect of varying concentrations of HTR-GA on the demulsification voltage of emulsions with different oil-to-water ratios.

Observational studies of pure emulsions (oil-water ratio of 80:20) over 7 days, as presented in Figure 3, indicate a minimal separation of oil in samples with 1% HTR-GA, a stark contrast to control samples. Further quantitative analysis, delineated in Figure 4, showcases a marked increase in emulsion-breaking voltage upon escalating HTR-GA concentrations, up to a point of saturation. This behavior is attributed to the formation of a composite interfacial membrane by HTR-GA adsorption, which fosters a weak gel matrix, impeding droplet coalescence and thus bolstering emulsion stability. The saturation point suggests an optimal concentration threshold for HTR-GA, beyond which no significant increase in stability is observed.

### 2.3. Impact of HTR-GA on Emulsion Rheology

To ascertain the efficacy of HTR-GA in modifying the viscosity profile of emulsions, comparative tests were conducted with a benchmark product, RHEMOD from Halliburton, DE, USA. Upon the addition of 1% HTR-GA and 1% RHEMOD to emulsions, followed by a thermal stress test involving 16 h of hot rolling at 220 °C, the emulsions’ rheological responses were measured across varied shear rates.

#### 2.3.1. Influence of HTR-GA on Emulsion Viscosity

**Figure 5:**
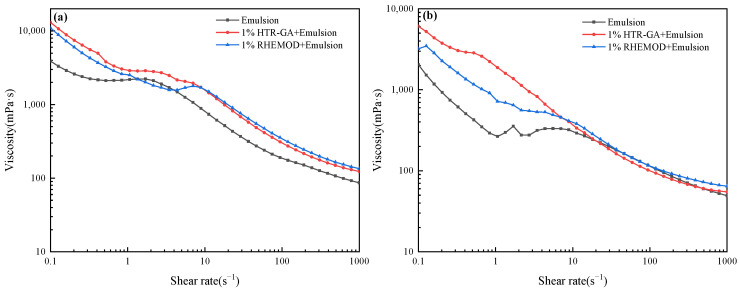
Rheological curves of different emulsions under various shear rates. (**a**) The rheological curve of the emulsion before aging. (**b**) The rheological curve of the emulsion following aging at 220 °C for 16 h.

The test emulsion’s composition, detailed, included 5# white oil, VERSAMUL, FACTANT, VERSACOAT, and CaCl_2_. Figure 5 shows the Rheological curves of different emulsions under various shear rates. As illustrated in Figure 5a, the rheological curves revealed that both HTR-GA and RHEMOD integrated emulsions manifested commendable shear-thinning characteristics, maintaining an elevated apparent viscosity at lower shear rates over the control sample. As depicted in Figure 5b, the HTR-GA enhanced emulsion exhibited superior low-shear viscosity relative to the RHEMOD emulsion, implying enhanced suspension capabilities for drill cuttings and weighting agents under static conditions or within annular spaces [33].

Conversely, at elevated shear rates encountered at the drill bit, the HTR-GA integrated emulsion displayed a reduced apparent viscosity, facilitating drilling efficiency and hydraulic power transmission. Notably, the superior performance of the HTR-GA-enhanced emulsion persisted even after prolonged exposure to high temperatures, underscoring HTR-GA’s robust high-temperature resistance and its potential to maintain optimal drilling fluid properties in extreme thermal environments.

#### 2.3.2. Effect of Gelling Agent on Thixotropy

**Figure 6:**
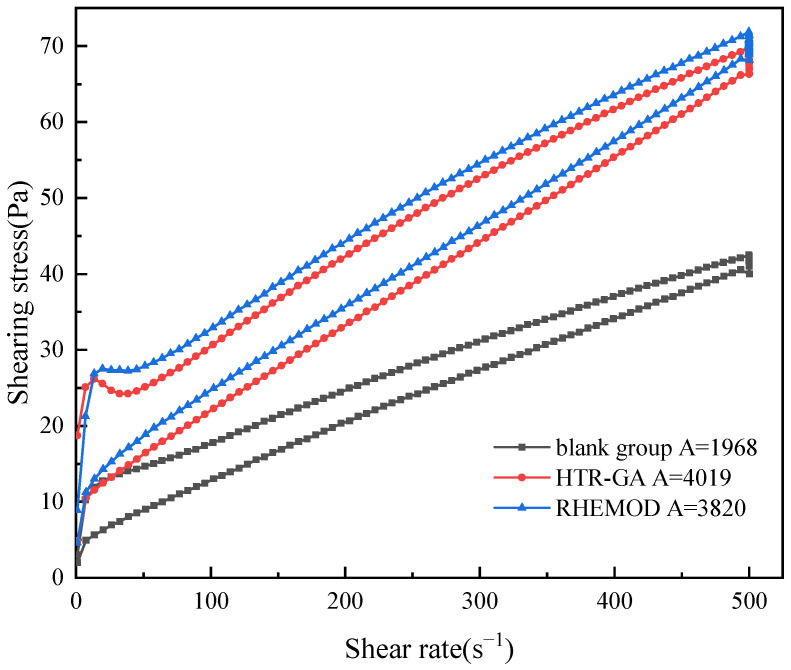
Thixotropic loops of different emulsions at shear rates ranging from 1 to 500 s⁻^1^.

The thixotropic properties of drilling fluids are crucial in maintaining wellbore stability and optimizing drilling processes. This study compares the thixotropic behavior of emulsions enhanced with 1% HTR-GA and 1% RHEMOD, after being subjected to a high-temperature endurance test (16 h at 220 °C). Post hot rolling, the thixotropic behavior was analyzed at 220 °C using a Haake rheometer, with results depicted in Figure 6. A pronounced hysteresis loop area, indicative of improved thixotropic behavior, was observed for both HTR-GA and RHEMOD-enhanced emulsions, signifying an enhanced ability to suspend solids and recover shear strength quickly after cessation of shear stress.

**Figure 7:**
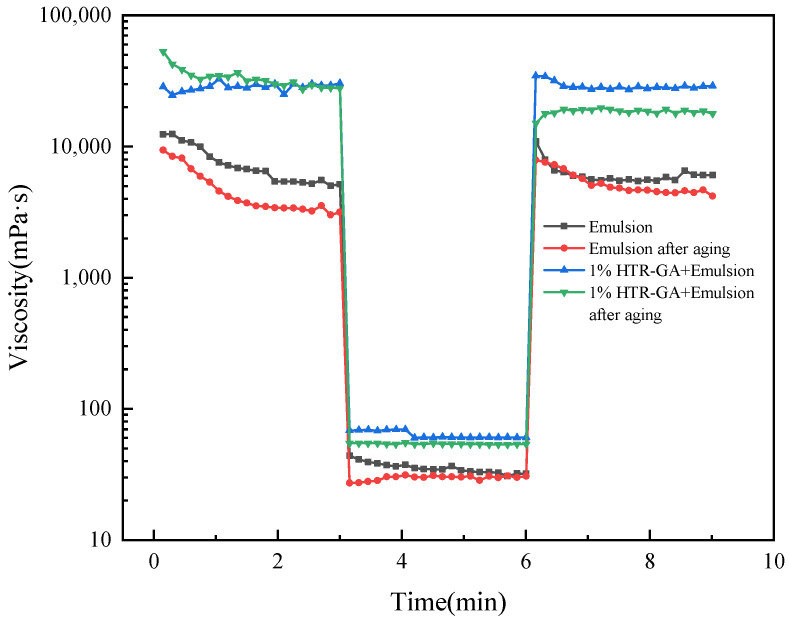
Three-stage thixotropy curves of the different emulsions.

Further insights into the thixotropic behavior are provided in Figure 7, which presents three-stage thixotropy curves at varying thickener concentrations. The emulsions demonstrated high low-shear viscosity, a precipitous viscosity reduction under high-shear, and swift viscosity recovery when shear rates were reduced, underscoring their thixotropic nature. Notably, the HTR-GA augmented emulsion showcased rapid gel strength recovery, exceeding that of the control sample, a property retained even after high-temperature aging. These findings attest to the fact that HTR-GA not only endows the emulsion with desirable thixotropic characteristics but also confers an enhanced gel recovery rate and strength, pivotal for the suspension of cuttings and reducing ECD during drilling operations. The study solidifies the position of HTR-GA as a viable gelling agent, capable of sustaining emulsion stability and functionality under extreme thermal conditions.

### 2.4. The Performance Evaluation of HTR-GA in Emulsions Containing Organic Clay

**Figure 8:**
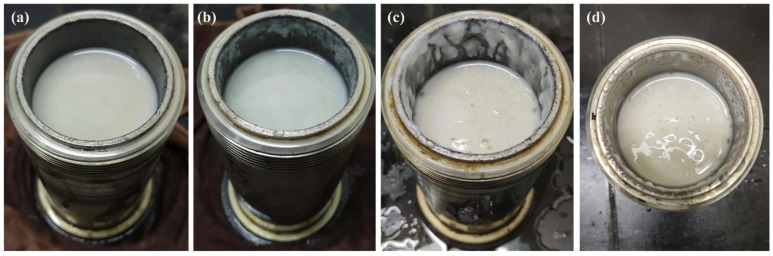
Visual inspection of emulsion samples post-aging: 1# emulsion after 16 h (**a**) and 32 h (**b**) of aging; 2# emulsion after 16 h (**c**) and 32 h (**d**) of aging.

HTR-GA has been demonstrated to augment the rheological attributes of oil-based drilling fluids devoid of organic clay. Nonetheless, the preponderance of such drilling fluid systems predominantly utilizes organic clay to establish their intricate spatial network structure, frequently supplemented with thickeners to fine-tune the rheological characteristics. It is, therefore, imperative to scrutinize the potential interplay between thickeners and organic clay, with a particular focus on ascertaining any synergistic actions that may collectively modulate the rheology of oil-based drilling fluids. Incorporating HTR-GA into emulsions replete with organic clay, we measured the resultant rheological behaviors using a Fann 35 six-speed rotational viscometer, as delineated in Table 2. The emulsions were aged at a temperature of 220 °C for durations of 16 and 32 h, with rheological assessments conducted at a temperature of 65 °C. The foundational emulsion composition comprised 5# white oil, a 25% solution of CaCl_2_, 3% VERSAMUL, 0.5% FACTANT, and 5% CaO, achieving an oil-to-water ratio of 80:20. Sample #1 represents the base emulsion, while Sample #2 is the emulsion with the addition of 1% HTR-GA. Figure 8 illustrates the post-aging states of emulsions #1 and #2 when the containers were opened after various intervals. This investigation underscores the significance of HTR-GA as a rheological enhancer in oil-based drilling fluids and the necessity of its comprehensive evaluation in systems containing organic clay.

The experimental outcomes revealed that before the initiation of the high-temperature aging process, Sample #2, when juxtaposed with Sample #1, manifested an enhancement in dynamic shear strength by 91%, and the storage modulus (G′) as well as the loss modulus (G″) were amplified by 200%. This enhancement led to a substantial amelioration of the emulsion’s rheological characteristics. Post aging at elevated temperatures, Sample #1 experienced a precipitous decline in rheological parameters, including dynamic shear strength, G′, and G″, which precipitated a significant degradation in rheological behavior, effectively compromising its ability to suspend and transport particulate matter. Moreover, the emulsion exhibited a noticeable reduction in viscosity upon opening after the aging period. In contrast, despite a reduction in the aforementioned rheological parameters after exposure to high-temperature aging, Sample #2 retained markedly better rheological properties than Sample #1. Additionally, the emulsion of Sample #2 preserved a stable state post-aging, characterized by an absence of oil separation at the upper layer, no sedimentation at the bottom, and no discernible change in viscosity. These observations ascertain that HTR-GA exhibits a synergistic relationship with emulsions containing organic clay, considerably enhancing the emulsion’s rheological attributes, particularly under conditions of high temperature.

### 2.5. Construction and Performance Evaluation of High-Temperature, High-Density Oil-Based Drilling Fluid System

#### 2.5.1. Selection of Base Oil, Matching Additives, and System Formulation

The 5# white oil was strategically chosen as the base oil. Its high flash and fire points ensure safety, while a high aniline point guards against damage to rubber elements in drilling equipment. The oil’s optimal viscosity aids in controlling drilling fluid rheology. Furthermore, its low environmental impact is noteworthy. Emulsifiers and wetting agents were carefully selected to fortify the emulsion’s stability and optimize the dispersion of weighting materials like barite. The system’s performance is a synergistic result of these components, with a specific focus on maintaining emulsion stability under extreme conditions, which is critical for the efficacy of the gelling agent HTR-GA. The final formulation achieved a balance between oil-to-water ratio and stability, with an optimized composition featuring a suite of additives and an 85:15 oil-to-water ratio. Through laboratory experimentation, the following formulation was selected for an anti-high-temperature, high-density oil-based drilling fluid system: 5# white oil combined with 3% VERSAMUL, 1% FACTANT, 3% VERSACOAT, 1% HTR-GA, and 25% CaCl_2_. The composition further includes 2% acrylic resin, 2% organic clay, 2% fine-grade calcium carbonate (800 mesh), 2% fine-grade calcium carbonate (2500 mesh), 5% calcium oxide, and barite to achieve a density of 2.2 g/cm^3^. The oil-to-water ratio was established at 85:15.

#### 2.5.2. Performance Evaluation of the High-Temperature, High-Density Oil-Based Drilling Fluid System

**Figure 9:**
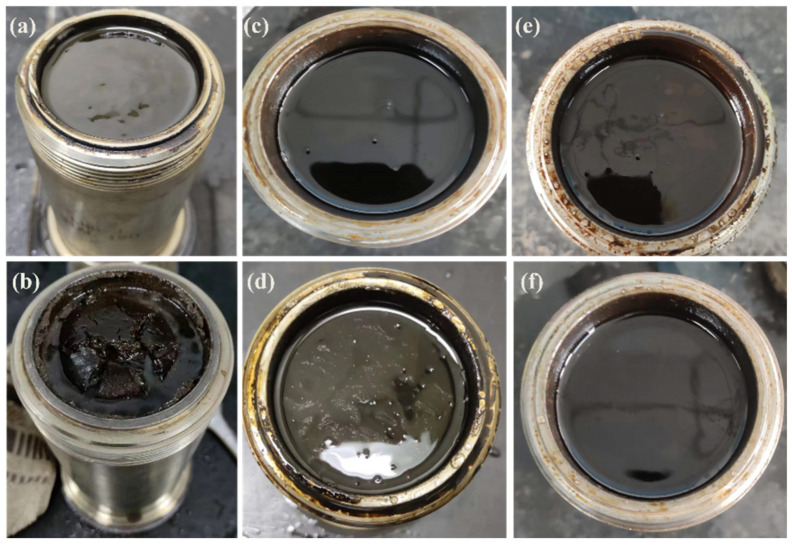
Post-aging condition of drilling fluid systems upon opening: for system #1 after 24 h (**a**) and 48 h (**b**), and for system #2 after 24 h (**c**), 48 h (**d**), 72 h (**e**), and 96 h (**f**).

The assessment was conducted under an aging temperature of 220 °C across multiple time points. Rheological measurements were taken at 65 °C. The system without a gelling agent, used for comparison, mirrored the optimized formulation but lacked HTR-GA. The results, encapsulated in Table 3 and visually represented in Figure 9, delineate the performance divergence over various aging intervals.

Before aging, the baseline fluid showcased excellent emulsion stability, but a marked rise in plastic viscosity post-aging suggested potential operational challenges. In stark contrast, the HTR-GA-enhanced fluid displayed not only superior initial stability but also maintained commendable rheological parameters even after prolonged thermal exposure, with key metrics like dynamic shear force and G″ exceeding the thresholds necessary for effective suspension and transport of solids. Centered on HTR-GA, a suite of complementary treatment agents was judiciously selected to construct an oil-based drilling fluid system capable of withstanding temperatures up to 220 °C and possessing a density of 2.2 g/cm^3^. This system exhibits favorable rheological properties; after aging at 220 °C for 24 h, the dynamic shear force exceeds 10 Pa, and the storage modulus (G′) is greater than 7 Pa. Moreover, after aging at 220 °C for 96 h, the dynamic shear force remains above 4 Pa, and the loss modulus (G″) reaches 7 Pa.

This empirical evidence underscores the success of formulating a high-temperature, high-density oil-based drilling fluid using HTR-GA as the central thickening agent. The system not only demonstrated excellent initial rheological characteristics but also sustained its performance criteria over extended high-temperature aging, thus showing promise for deployment in deep and ultra-deep drilling operations and maintaining long-term high-temperature stability suitable for practical application.

#### 2.5.3. Evaluation of the Contamination Resistance of Oil-Based Drilling Fluids

In the context of drilling operations in deep and ultra-deep wells, practitioners frequently encounter intricate geological formations, inclusive of high-pressure aquifers and strata comprising gypsum. Under such scenarios, contaminants such as saline solutions and gypsum have the propensity to markedly impair the efficacy of oil-based drilling fluids, particularly when subjected to cycles of elevated temperatures. A systematic assessment was conducted to ascertain the contaminant resistance capabilities of the drilling fluid system, focusing on analyzing both rheological attributes and electrochemical stability. This evaluation entailed subjecting the fluid system to a controlled aging process under varied conditions, incorporating additions of freshwater, saline solution (concentration: 180 g/L NaCl), and gypsum. The ensuing results are methodically tabulated in Table 4, which delineates the specific contamination factors as the respective quantities incorporated per liter of the drilling fluid. Notably, the data in Table 4 illustrates that the fluid system efficaciously sustains its rheological integrity and electrochemical stability under the experimental conditions of 15% freshwater, 10% saline solution, and 10% CaSO_4_. This demonstrates a pronounced resilience of the system against contaminant infiltration.

### 2.6. Mechanism Analysis

#### 2.6.1. Interface Membrane Property Examination

**Figure 10:**
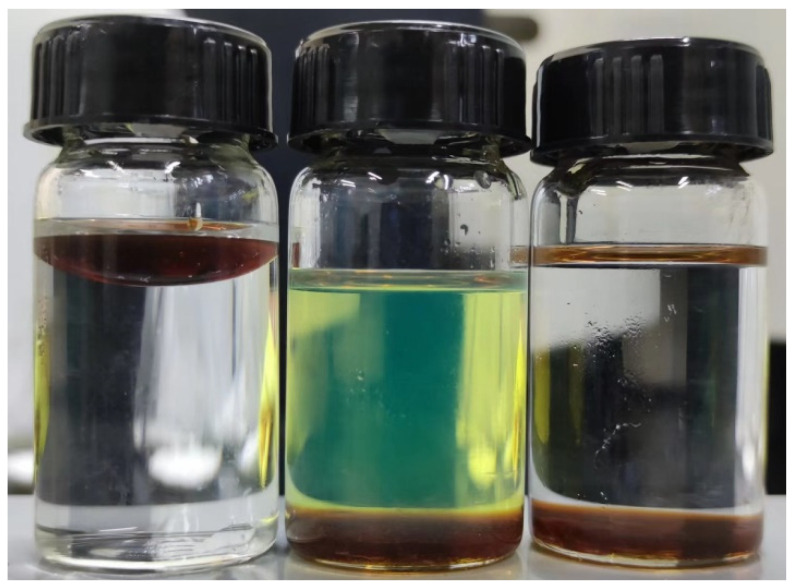
Solubility experiments of HTR-GA in water, diesel, and kerosene.

**Figure 11:**
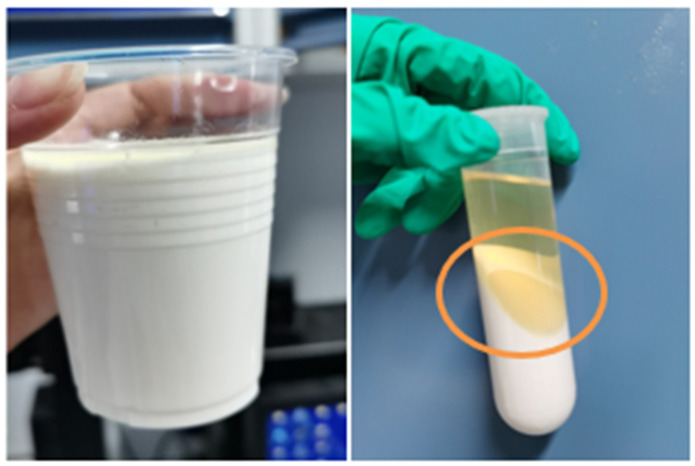
Precipitation of HTR-GA at the phase interface following centrifugation.

A comprehensive analysis was conducted to decipher the mechanism behind the performance of the synthesized HTR-GA. Being an amphiphilic block copolymer, HTR-GA exhibits a natural propensity to adsorb at the oil-water interface. Solubility tests, depicted in Figure 10, showed limited solubility of HTR-GA in white oil and diesel, revealing that it is not wholly oil-soluble. However, when integrated into an emulsion system and subjected to vigorous stirring, HTR-GA dispersed effectively, and subsequent centrifugation confirmed its preferential accumulation at the phase interface, as shown in Figure 11. Within the orange circle is HTR-GA adsorbed at the oil-water interfacial boundary.

Further investigation into the effects of HTR-GA on the interfacial properties of the emulsion was conducted using a high-temperature, high-pressure interfacial tension meter. The data, compiled in Table 5, indicated a marginal rise in oil-water interfacial tension with increasing HTR-GA concentrations. Considering the critical role of interfacial tension and membrane strength in emulsion stability, and in light of observations from Figure 3, Figure 4, and Table 5, it is postulated that HTR-GA reinforces the strength of the oil-water interface membrane, enhancing the emulsion’s stability. The findings suggest that HTR-GA’s mode of action involves adsorption and fortification of the oil-water interface, contributing to the overall stability and functional efficacy of the drilling fluid system.

#### 2.6.2. Evaluation of Emulsion Aggregation Morphology

**Figure 12:**
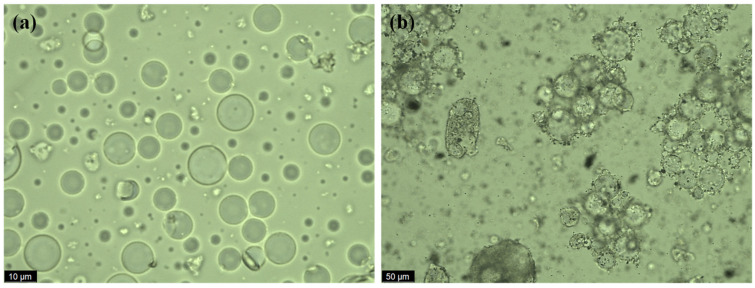
Microstructural morphology of emulsions post High-Speed shearing and subsequent resting: (**a**) Morphology of pure emulsion after 30 s of rest post-high-speed shearing. (**b**) Morphology of pure emulsion with HTR-GA after 30 s of rest post-high-speed shearing.

The microscopic examination of the emulsion’s morphology, post-high-speed shear with HTR-GA addition, was conducted using optical microscopy, with findings depicted in Figure 12. The observed emulsion, comprising a specific formulation including 5# white oil and HTR-GA, demonstrated droplets aggregated into gel-like structures reminiscent of “grape clusters.” These clusters form a complex spatial network, thereby augmenting the emulsion’s shear strength.

**Figure 13:**
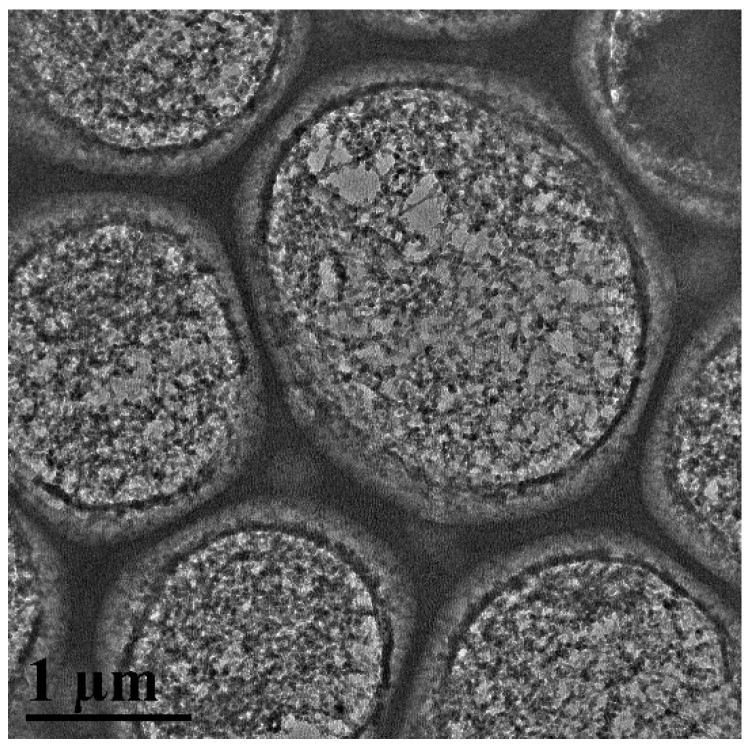
Accumulation of elastic interfacial films (Cryo-Electron Microscopy).

Further microstructural insights were gleaned through cryo-electron microscopy, as exhibited in Figure 13. Post HTR-GA addition, a denser and more viscoelastic interface membrane was observed, supporting the formation of a robust gel structure at the interface. Macroscopically, these findings correlate with heightened rheological parameters such as shear force, G′, and G″, indicating an enhanced ability to suspend and transport solids within the drilling fluid.

**Figure 14:**
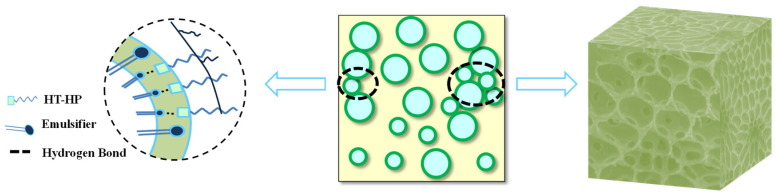
Schematic illustration of the mechanism of action of HTR-GA.

Figure 14 delineates the mechanism by which HTR-GA modulates emulsion rheology. At low shear rates, HTR-GA synergistically interacts with emulsifier molecules, forging a composite elastic interface membrane that connects droplets to form a cohesive microgel structure. This structure contributes to an organized spatial network within the drilling fluid, characterized by strength, swift recovery, and pronounced thixotropy, thus increasing the viscosity and shear strength under static conditions. Conversely, at high shear rates, the disruption of non-covalent bonds leads to the disintegration of the weak gel structure, and due to HTR-GA’s low molecular weight and reduced internal friction, the viscosity increment is restrained. This dual functionality of HTR-GA underscores its potential to enhance drilling fluid performance across a range of operational conditions.

## 3. Conclusions

(1) An amphiphilic multiblock polymer synthesized from dimeric fatty acids and polyether amines has been developed as a high-temperature gelling agent (HTR-GA) for oil-based drilling fluids. It exhibits an initial decomposition temperature of 374 °C, indicating excellent thermal stability.
(2) HTR-GA enhances the electron stability of emulsions and effectively improves their rheological properties at high temperatures, augmenting their shear-thinning capabilities.
(3) Centering on HTR-GA, a high-temperature-resistant oil-based drilling fluid system has been formulated, capable of withstanding temperatures up to 220 °C and densities up to 2.2g/cm³, while maintaining favorable rheological properties. This system demonstrates significant application potential.
(4) HTR-GA adsorbs at multiple sites on the oil-water interfaces of various emulsions, thereby enhancing the interfacial film strength and increasing the agglomeration of adjacent droplets, leading to the formation of a gel network structure that can extend locally or throughout the emulsion.
(5) A gelling agent has been developed for regulating the rheological properties of drilling fluids under varying temperature conditions, including high and low-temperature cycles, enhancing the adaptability of drilling fluids in diverse thermal environments.

## 4. Materials and Methods

### 4.1. Reagents and Instruments

The primary experimental reagents were sourced with poly fatty acids (95% purity) and polyether amine procured from Aladdin, based in Shanghai, China. Agents for treating oil-based drilling fluids were furnished by industry leaders M-I SWACO and Baroid. The 5# base oil, utilized for the experimental fluid matrix, was obtained from Maoming Petrochemical, characterized by a density of 0.88 g/cm^3^ at 25 °C and an apparent viscosity of 24.4 mPa·s at a shear rate of 1021.8 s⁻^1^.

Instrumentation was pivotal to the study, encompassing a Leica DM4M optical microscope from Germany for microstructural observation, a JEOL (Akishima, Japan) JEM-1400 transmission electron microscope from Japan for ultrastructural analysis, and a DTCA21 spinning drop tensiometer from Data Physics Inc. (Riverside, CA, USA) for interfacial tension measurements. Molecular characterization was conducted using a Magna-IR 560 spectrometer from Nicolet Inc., Green Bay, WI, USA, and thermogravimetric analysis was performed with a NETZSCH STA 499 F5 analyzer from Selb, Germany. Rheological properties were measured with a Haake MARS III rheometer from Thermo Fisher Scientific (Waltham, MA, USA), while emulsion stability was determined using a Fann 23D demulsification voltage tester and a Fann 35 six-speed rotational viscometer, both from FANN, Houston, TX, USA. High-speed mixing was achieved with a GJSS-B12K variable-frequency agitator and thermal treatments were conducted in a GW300 roller heating furnace, both acquired from Qingdao Tongchun Petroleum Instrument Co., Ltd. (Qingdao, China).

### 4.2. Synthesis of HTR-GA

**Figure 15:**
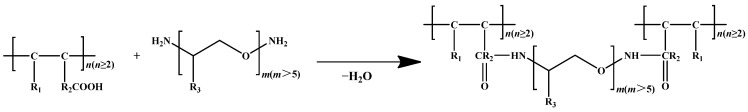
Synthetic Reaction Scheme of HTR-GA.

The synthetic route for HTR-GA was conducted via the amidation reaction of poly fatty acids with polyether amines. A meticulously dried three-neck flask was outfitted on an iron stand, equipped with a mechanical stirrer, a water separator, and an inlet for nitrogen gas to create an inert atmosphere. The reactants were introduced into the flask at a stoichiometric ratio of 1:2 (poly fatty acids to polyether amines), and an inert environment was established by purging with nitrogen for 10 min before the onset of the reaction. The mixture was then subjected to an oil bath preheated to 150 °C, under continuous stirring at 250 rpm for 4 h, yielding a dark brown viscous product indicative of the HTR-GA thickener. The synthetic route for HTR-GA is illustrated in Figure 15.

### 4.3. Preparation of Fluids

#### 4.3.1. Preparation of Pure Emulsions

The assembly of pure emulsions necessitated precise oil-water ratios. The process commenced with the addition of a specified volume of 3# base oil into a high-speed stirring apparatus. Subsequently, the surfactant Span80, followed by a 25% CaCl_2_ aqueous solution, was incorporated under intense stirring to achieve homogeneity. An analogous procedure was employed for the 2# emulsion, albeit with VERSAMUL and FACTANT as the emulsifying agents.

#### 4.3.2. Preparation of Emulsions Containing Organic Clay

An identical approach was adopted for emulsions with organic clay, with the inclusion of VERSAMUL, FACTANT, and organic clay, followed by a 25% CaCl_2_ solution, each subjected to rigorous stirring to ensure a thorough mix.

#### 4.3.3. Preparation of Oil-Based Drilling Fluids

Oil-based drilling fluids were prepared by combining 3# base oil and various emulsifiers, heated to enhance solubility, followed by the sequential addition of slurry constituents such as calcium oxide and organic clay. The mixture was then agitated at high speed, after which barite was added to attain the desired density, concluding the preparation process.

### 4.4. Evaluation Methods

#### 4.4.1. FTIR

Utilizing the Magna-IR560 spectrometer, infrared spectral data about HTR-GA were acquired for the elucidation of functional group characteristics. The HTR-GA sample was finely pulverized in conjunction with potassium bromide (KBr), thoroughly amalgamated, and subsequently compressed into thin films. The Fourier transform infrared (FTIR) absorption spectrum of the HTR-GA was meticulously recorded across the spectral domain extending from 4000 to 400 cm^−1^ employing the aforementioned FTIR spectrometric technique.

#### 4.4.2. TGA

Employing the NETZSCH STA499F5 thermogravimetric analyzer, the thermal properties of the high-temperature thickening agent HTR-GA were investigated. This analysis facilitated the acquisition of thermogravimetric (TG) and differential thermogravimetric (DTG) curves of the sample. The experimental conditions were maintained under a nitrogen atmosphere, with the temperature parameter set to escalate from ambient level to 500 °C at a uniform heating rate of 15 °C/min.

#### 4.4.3. GPC

Gel Permeation Chromatography (GPC), a sophisticated analytical technique, has been utilized for the determination of the relative molecular weights of the samples under study. In this procedure, Tetrahydrofuran (THF) serves as the eluent, while polystyrene is employed as the calibration standard. The chromatographic separation was conducted under meticulously controlled conditions, maintaining the column temperature at a stable 30 °C, and regulating the eluent flow rate at a precise 1 mL/min.

#### 4.4.4. Rheological Curve Testing

The viscosities of the pure emulsion and the HTR-GA-laden emulsion samples were quantified via the Haake MARS III rheometer. The instrument was configured with a parallel plate geometry, specifying a gap of 0.047 mm between the plates. The shear rate was systematically varied over a wide range, spanning from 0.1 to 1000 s^−1^. All measurements were conducted under a rigorously controlled temperature of (25.0 ± 0.1) °C to ensure isothermal conditions throughout the experimental procedure.

#### 4.4.5. Thixotropic Testing

The thixotropic behavior of the emulsion was investigated utilizing the Haake MARS III rheometer. A thixotropic loop was delineated employing a continuous rotation protocol. The experimental regime involved subjecting the sample to an ascending shear rate that increased from 1 s^−1^ to 500 s^−1^, followed by a descending shear rate from 500 s^−1^ back to 1 s^−1^. This cycle was executed over 20 min, uninterrupted, to ensure the integrity of the thixotropic loop analysis.

#### 4.4.6. Three-Interval Thixotropy Test

The “rest-shear-rest” triphasic thixotropic characteristics of the emulsion samples were measured with the Haake MARS III rheometer, utilizing a parallel-plate rotor. Initially, the emulsion sample underwent a low-speed shear at 0.1 s^−1^ for 3 min, which served as the first rest phase. This was followed by a high-speed shear at 1000 s^−1^ for another 3 min, representing the shear phase. Finally, the sample was subjected once more to a low-speed shear at 0.1 s^−1^ for 3 min, constituting the second rest phase. The entirety of this triphasic procedure was performed at a controlled temperature of (25.0 ± 0.1) °C to maintain thermal consistency.

#### 4.4.7. High-Temperature Aging Experiment

The prepared fluid was hermetically sealed within an aging cell, which was then placed in a GW300-X type high-temperature roller oven to undergo thermal rolling (the specific temperature and duration were adjusted according to the requirements). Following the thermal treatment, the aging cell was cooled down and subsequently opened. The drilling fluid was then poured into a high-shear mixing cup and stirred at a velocity of 12,000 rpm for 30 min. After this high-shear mixing process, further evaluations of the drilling fluid’s properties were conducted.

#### 4.4.8. Emulsion Stability Test

The emulsion-breaking voltage of the drilling fluid was quantified using a DWY-2-type electrical stability tester. The temperature was maintained at 65 °C during the tests. For each sample, the measurement was performed thrice, and the mean value of these trials was calculated to ensure accuracy. Before each measurement, the instrument was calibrated to guarantee precision. After each measurement, the probe was meticulously cleaned to prevent any cross-contamination or residue effects on subsequent readings. The emulsion-breaking voltage is indicative of the voltage at which the droplets within the emulsion are disrupted by the peak voltage. Generally, a higher emulsion-breaking voltage suggests greater stability within the drilling fluid system.

#### 4.4.9. Rheological Performance Testing

Finally, the comprehensive rheological performance of the drilling fluids was appraised using a rotational viscometer, adhering to API standards to determine parameters [34] such as apparent viscosity (AV), plastic viscosity (PV), yield point (YP), G′, and G″.

(1) $$AV=0.5\emptyset_{600}$$

(2) $$PV=\emptyset_{600}-\emptyset_{300}$$

(3) $$YP=0.511\left(\emptyset_{300}-PV\right)$$

(4) $$G^{’}=0.511\emptyset_{3}\left(10\;s\right)$$

(5) $$G^{\prime \prime }=0.511\emptyset_{3}\left(10\;min\right)$$

Herein, $\emptyset_{3}\left(10\;s\right)$ refers to the maximum value of $\emptyset_{3}$ recorded for the drilling fluid after it has been thoroughly stirred and then allowed to stand undisturbed for 10 s. Meanwhile, $\emptyset_{3}\left(10\;min\right)$ denotes the maximum value of $\emptyset_{3}$ ascertained after the drilling fluid has been adequately mixed and subsequently left to rest for 10 min.

#### 4.4.10. Interfacial Tension Testing

The interfacial tension testing is an essential step in understanding the interactions at the paraffin-water interface in the presence of SPAN-80 and the synthesized HTR-GA. Utilizing a DTCA21 spinning drop tensiometer, the interfacial tension measurements were carried out at a controlled temperature of 25 °C, which is vital for determining the emulsifying efficiency of HTR-GA.

#### 4.4.11. Cryo-Transmission Electron Microscopy

To elucidate the microstructure of emulsions augmented with HTR-GA, cryo-scanning electron microscopy was employed. Samples were prepared in a humidity-controlled environment and flash-frozen in liquid ethane to preserve their native state. The cryogenically stabilized samples were then examined under a JEOL JEM-1400 transmission electron microscope. This technique allows for the observation of emulsions in a state closest to their natural aqueous environment, thus providing invaluable insights into the microstructural efficacy of HTR-GA.

#### 4.4.12. Optical Microscopy

For the detailed visualization of the emulsion’s microscopic morphology, a Leica DM4M optical microscope was utilized. Samples were adequately diluted with base oil to facilitate clarity and then meticulously positioned between glass slides for observation. Capturing images in bright-field mode provided a comprehensive understanding of the emulsion morphology, further affirming the structural influence of HTR-GA.

## Figures and Tables

**Table 1:** Molecular weight characterization of the synthesized HTR-GA.

Mn/ Daltons	Mw/ Daltons	Mp/ Daltons	Mz/ Daltons
19,547	40,263	37,201	66,916

**Table 2:** Performance evaluation of HTR-GA in organic clay-containing emulsions.

Type	Aging Duration	AV/	PV/	YP/	Ф6/Ф3	G′(G″)/	ES/
		mPa·s	mPa·s	Pa		Pa(Pa)	V
Sample #1	0 h	20.5	15	5.6	4/3	2/3	589
	16 h	13	11	2.0	3/2	1.5/2	459
	32 h	10	9	1.0	2/1	0.5/1	408
Sample #2	0 h	30.5	20	10.7	11/10	6/9	896
	16 h	23	15	8.2	10/9	5/7.5	824
	32 h	22.5	16	6.6	8/7	4.5/7	613

**Table 3:** Comparative analysis of oil-based drilling fluid properties at varied aging durations.

Type	Aging Duration	AV/ mPa·s	PV/ mPa·s	YP/ Pa	Ф6/Ф3	G′(G″)/ Pa(Pa)	ES/ V
#1	0 h	49	44	5.11	8/6	3.5/4	1352
	24 h	75	60	15.33	24/22	10.5/22	2011
#2	0 h	52	39	13.29	12/11	7.5/12	1865
	24 h	43.5	33	10.73	11/10	7/12	1768
	48 h	41	32	9.20	10/9	6/10	1592
	72 h	37	32	5.11	8/7	5.5/8	1235
	96 h	35.5	31	4.60	6/5	4.5/7	1086

**Table 4:** Experiment on the Contamination Resistance of Oil-Based Drilling Fluids.

Contamination Factor	PV/ mPa·s	YP/ Pa	G′(G″)/ Pa(Pa)	ES/ V
Before pollution	33	10.73	7 (12)	1840
150 mL water	55	16.32	8 (15)	856
100 mL saline	64	18.36	9.5 (16)	765
100 g CaSO_4_	72	7.14	8.5 (15)	1697

**Table 5:** The impact of HTR-GA on Oil-Water interfacial tension.

Span-80/ %	HTR-GA/ %	Interfacial Tension/ mN/m
4	0	0.257
4	0.5	0.274
4	1	0.285

## Data Availability

Data is contained within the article.
